# Diaqua­bis[6-(3,5-dimethyl-1*H*-pyrazol-1-yl)picolinato-κ^3^ *N*,*N*′,*O*](nitrato-κ^2^ *O*,*O*′)lanthanum(III) monohydrate

**DOI:** 10.1107/S1600536808001955

**Published:** 2008-01-25

**Authors:** Zhao Kai, Xian-Hong Yin, Feng Yu, Zhu Jie, Cui-Wu Lin

**Affiliations:** a College of Chemistry and Ecological Engineering, Guangxi University for Nationalities, Nanning 530006, People’s Republic of China; b College of Chemistry and Chemical Engineering, Guangxi University, Nanning 530004, People’s Republic of China

## Abstract

In the title complex, [La(C_11_H_10_N_3_O_2_)_2_(NO_3_)(H_2_O)_2_]·H_2_O, the La atom is coordinated by four N atoms and six O atoms derived from two 6-(3,5-dimethyl-1*H*-pyrazol-1-yl)picolinate ligands, one nitrate anion and two water mol­ecules. The mol­ecules are linked together *via* hydrogen bonds involving the water mol­ecules, forming a three-dimensional network.

## Related literature

For related literature, see: Zhao *et al.* (2007 ▶); Yin *et al.* (2007 ▶).
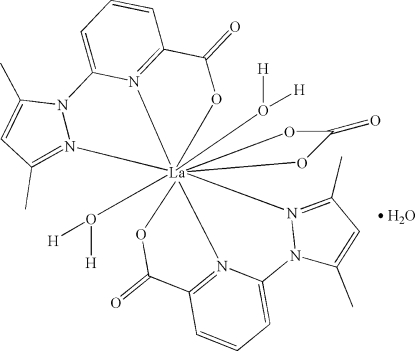


## Experimental

### 

#### Crystal data

[La(C_11_H_10_N_3_O_2_)_2_(NO_3_)(H_2_O)_2_]·H_2_O
*M* *_r_* = 687.41
Monoclinic, 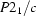

*a* = 17.396 (2) Å
*b* = 15.0270 (18) Å
*c* = 10.1607 (13) Å
β = 94.737 (2)°
*V* = 2647.0 (6) Å^3^
*Z* = 4
Mo *K*α radiation
μ = 1.68 mm^−1^
*T* = 298 (2) K
0.46 × 0.45 × 0.40 mm

#### Data collection

Siemens SMART CCD area-detector diffractometer
Absorption correction: multi-scan (*SADABS*; Sheldrick, 1996 ▶) *T*_min_ = 0.512, *T*_max_ = 0.553 (expected range = 0.473–0.510)
13524 measured reflections
4656 independent reflections
3941 reflections with *I* > 2σ(*I*)
*R*_int_ = 0.022

#### Refinement

*R*[*F* ^2^ > 2σ(*F* ^2^)] = 0.025
*wR*(*F* ^2^) = 0.076
*S* = 1.01
4656 reflections
361 parameters
H-atom parameters constrained
Δρ_max_ = 0.68 e Å^−3^
Δρ_min_ = −0.55 e Å^−3^

### 

Data collection: *SMART* (Siemens, 1996 ▶); cell refinement: *SAINT* (Siemens, 1996 ▶); data reduction: *SAINT*; program(s) used to solve structure: *SHELXS97* (Sheldrick, 2008 ▶); program(s) used to refine structure: *SHELXL97* (Sheldrick, 2008 ▶); molecular graphics: *SHELXTL* (Sheldrick, 2008 ▶); software used to prepare material for publication: *SHELXTL*.

## Supplementary Material

Crystal structure: contains datablocks I, global. DOI: 10.1107/S1600536808001955/hg2369sup1.cif

Structure factors: contains datablocks I. DOI: 10.1107/S1600536808001955/hg2369Isup2.hkl

Additional supplementary materials:  crystallographic information; 3D view; checkCIF report

## Figures and Tables

**Table 1:** Hydrogen-bond geometry (Å, °)

*D*—H⋯*A*	*D*—H	H⋯*A*	*D*⋯*A*	*D*—H⋯*A*
O8—H8*B*⋯O10^i^	0.85	2.13	2.918 (5)	154
O8—H8*C*⋯O4^ii^	0.85	1.91	2.713 (4)	158
O9—H9*B*⋯O2^iii^	0.85	1.96	2.731 (4)	151
O9—H9*B*⋯N1	0.85	2.46	2.878 (4)	112
O10—H10*C*⋯N6^iv^	0.85	2.49	3.156 (5)	136
O10—H10*D*⋯O9^v^	0.85	2.24	2.977 (5)	146
O10—H10*D*⋯O1^iv^	0.85	2.46	2.913 (4)	114

Symmetry codes: (i) 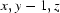; (ii) 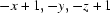; (iii) 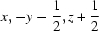; (iv) 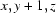; (v) 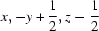.

**Fig. 1.**
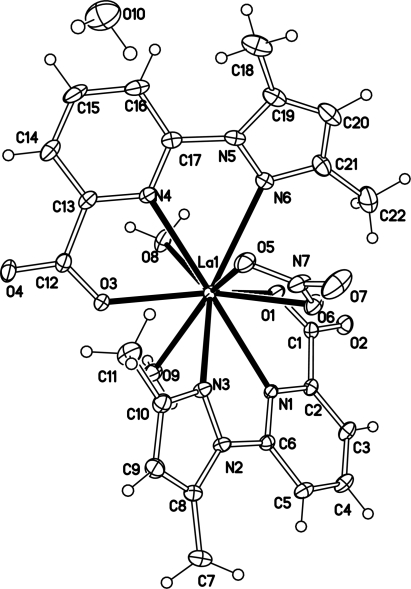
The structure of the title compound (I) showing 50% probability displacement ellipsoids and the atom-numbering scheme.

**Fig. 2.**
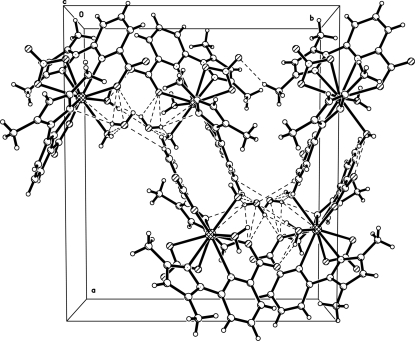
Crystal packing of (I) showing the hydrogen bonded interactions as dashed lines.

## supplementary crystallographic information

### Comment

Recently we reported the crystal structures of bis(6-(3,5-dimethyl-1*H*-pyrazol-1-yl)picolinato)zinc(II) trihydrate (Yin *et al.*, 2007) and bis[3-chloro-6-(3,5-dimethyl-1*H*-pyrazol-1-yl)picolinato]cobalt(II) 2.5- hydrate (Zhao *et al.*, 2007). As a continuation of these investigations, we report in this paper the crystal structure of Nitrato-diaqua-bis(6-(3,5-dimethyl-1*H*-pyrazol-1-yl)) picolinato)lanthanum(III) monohydrate.

The asymmetric unit of the title structure consists of the central mononuclear lanthanum(III) complex and one uncoordinated water molecule. The La atom is ten-coordinated by four N atoms and six O atoms derived from two 6-(3,5-dimethyl-1*H*-pyrazol-1-yl)picolinate ligands (DPP), one bidentate nitrate anion and two water molecules that define a pseudotricapped trigonal environment for the lanthanum atom. The angles around La(III) atom range from 47.99 (8) to 144.42 (10)°, the La—O distances range from 2.452 (2) to 2.676 (3) Å, the La—N distances range is from 2.688 (3) to 2.811 (3) Å.

In the crystal structure, the oxygen atoms contribute to the formation of intermolecular hydrogen bonds involving the water molecules; three water molecules and three DDP O atoms form a rings *via* intermolecular H—O···H hydrogen bonds. A great number of hydrogen contacts link the complex into a three-dimensional network. (Fig. 2; for symmetry codes see Table 2).

### Experimental

6-(3,5-dimethyl-1*H*-pyrazol-1-yl)picolinic acid, and La(NO_3_)_3_. 6H_2_O were available commercially and were used without further purification. Equimolar 6-(3,5-dimethyl-1*H*-pyrazol-1-yl)picolinic acid (1 mmol, 217 mg) was dissolved in anhydrous ethyl alcohol (AR,99.9%) (15 ml). The mixture was stirred to give a clear solution, To this solution was added La(NO_3_)_3_. 6H_2_O (0.33 mmol, 144 mg) in anhydrous alcohol (10 ml). After keeping the resulting solution in air to evaporate about half of the solvents, colorless blocks of the title complex were formed. The crystals were isolated, washed with alcohol three times (Yield 75%). Elemental analysis: found: C, 38.24; H, 3.91; N, 14.16%; calc. for C_22_H_26_LaN_7_O_10_: C, 38.44; H, 3.81; N, 14.26%.

### Refinement

H atoms on C atoms were positoned geometrically and refined using a riding model with C—H = 0.96Å and *U*_iso_(H) = 1.2*U*_eq_(C). The water H atoms were located in difference Fourier maps and the O—H distances were constrained 0.85 Å, with *U*_iso_(H) = 1.2*U*_eq_(O).

### Crystal data

**Table:**

[La(C_11_H_10_N_3_O_2_)_2_(NO_3_)(H_2_O)_2_]·H_2_O	*F*_000_ = 1376
*M**_r_* = 687.41	*D*_x_ = 1.725 Mg m^−^^3^
Monoclinic, *P*2_1_/*c*	Mo *K*α radiation λ = 0.71073 Å
Hall symbol: -P 2ybc	Cell parameters from 8663 reflections
*a* = 17.396 (2) Å	θ = 2.4–28.3º
*b* = 15.0270 (18) Å	µ = 1.68 mm^−^^1^
*c* = 10.1607 (13) Å	*T* = 298 (2) K
β = 94.737 (2)º	Block, colorless
*V* = 2647.0 (6) Å^3^	0.46 × 0.45 × 0.40 mm
*Z* = 4	

### Data collection

**Table:**

Siemens SMART CCD area-detector diffractometer	4656 independent reflections
Radiation source: fine-focus sealed tube	3941 reflections with *I* > 2σ(*I*)
Monochromator: graphite	*R*_int_ = 0.022
*T* = 298(2) K	θ_max_ = 25.0º
φ and ω scans	θ_min_ = 1.8º
Absorption correction: multi-scan(SADABS; Sheldrick, 1996)	*h* = −19→20
*T*_min_ = 0.512, *T*_max_ = 0.553	*k* = −17→13
13524 measured reflections	*l* = −12→12

### Refinement

**Table:**

Refinement on *F*^2^	Secondary atom site location: difference Fourier map
Least-squares matrix: full	Hydrogen site location: inferred from neighbouring sites
*R*[*F*^2^ > 2σ(*F*^2^)] = 0.025	H-atom parameters constrained
*wR*(*F*^2^) = 0.077	*w* = 1/[σ^2^(*F*_o_^2^) + (0.0425*P*)^2^ + 3.0256*P*] where *P* = (*F*_o_^2^ + 2*F*_c_^2^)/3
*S* = 1.01	(Δ/σ)_max_ = 0.001
4656 reflections	Δρ_max_ = 0.68 e Å^−^^3^
361 parameters	Δρ_min_ = −0.55 e Å^−^^3^
Primary atom site location: structure-invariant direct methods	Extinction correction: none

### Special details

**Table:**

Geometry. All e.s.d.'s (except the e.s.d. in the dihedral angle between two l.s. planes) are estimated using the full covariance matrix. The cell e.s.d.'s are taken into account individually in the estimation of e.s.d.'s in distances, angles and torsion angles; correlations between e.s.d.'s in cell parameters are only used when they are defined by crystal symmetry. An approximate (isotropic) treatment of cell e.s.d.'s is used for estimating e.s.d.'s involving l.s. planes.
Refinement. Refinement of *F*^2^ against ALL reflections. The weighted *R*-factor *wR* and goodness of fit *S* are based on *F*^2^, conventional *R*-factors *R* are based on *F*, with *F* set to zero for negative *F*^2^. The threshold expression of *F*^2^ > σ(*F*^2^) is used only for calculating *R*-factors(gt) *etc*. and is not relevant to the choice of reflections for refinement. *R*-factors based on *F*^2^ are statistically about twice as large as those based on *F*, and *R*- factors based on ALL data will be even larger.

### Fractional atomic coordinates and isotropic or equivalent isotropic displacement parameters (Å^2^)

**Table:**

	*x*	*y*	*z*	*U*_iso_*/*U*_eq_	
La1	0.725615 (10)	−0.033374 (12)	0.360394 (18)	0.02756 (8)	
N1	0.86114 (15)	−0.10885 (18)	0.4512 (3)	0.0267 (6)	
N2	0.88283 (16)	0.0047 (2)	0.6034 (3)	0.0325 (6)	
N3	0.82454 (16)	0.05236 (19)	0.5339 (3)	0.0342 (7)	
N4	0.59938 (17)	0.0651 (2)	0.2758 (3)	0.0353 (7)	
N5	0.6301 (2)	0.0557 (2)	0.0593 (3)	0.0460 (8)	
N6	0.6814 (2)	−0.0130 (3)	0.0897 (3)	0.0489 (9)	
N7	0.83143 (19)	0.0967 (2)	0.2242 (4)	0.0497 (9)	
O1	0.75996 (14)	−0.17799 (17)	0.2701 (3)	0.0428 (6)	
O2	0.83910 (15)	−0.28912 (17)	0.2271 (3)	0.0442 (6)	
O3	0.64201 (14)	0.02652 (17)	0.5260 (3)	0.0391 (6)	
O4	0.53914 (16)	0.0940 (2)	0.5969 (3)	0.0562 (8)	
O5	0.76750 (16)	0.12248 (19)	0.2609 (3)	0.0500 (7)	
O6	0.84704 (16)	0.01423 (19)	0.2392 (3)	0.0479 (7)	
O7	0.87668 (19)	0.1474 (3)	0.1778 (5)	0.0911 (14)	
O8	0.60430 (15)	−0.13605 (19)	0.3366 (3)	0.0529 (8)	
H8B	0.5958	−0.1549	0.2578	0.064*	
H8C	0.5645	−0.1090	0.3590	0.064*	
O9	0.72302 (15)	−0.12026 (18)	0.5879 (3)	0.0466 (7)	
H9B	0.7684	−0.1352	0.6167	0.056*	
H9C	0.6957	−0.1670	0.5773	0.056*	
O10	0.6289 (2)	0.7859 (2)	0.0804 (4)	0.0771 (10)	
H10C	0.6333	0.8333	0.1260	0.093*	
H10D	0.6685	0.7544	0.1017	0.093*	
C1	0.8231 (2)	−0.2207 (2)	0.2862 (3)	0.0329 (8)	
C2	0.88316 (19)	−0.1827 (2)	0.3894 (3)	0.0303 (7)	
C3	0.9541 (2)	−0.2219 (3)	0.4173 (4)	0.0423 (9)	
H3	0.9677	−0.2731	0.3733	0.051*	
C4	1.0046 (2)	−0.1830 (2)	0.5129 (4)	0.0458 (10)	
H4	1.0528	−0.2082	0.5340	0.055*	
C5	0.9835 (2)	−0.1071 (3)	0.5771 (4)	0.0394 (9)	
H5	1.0172	−0.0798	0.6406	0.047*	
C6	0.91020 (18)	−0.0727 (2)	0.5436 (3)	0.0293 (7)	
C7	0.9641 (3)	0.0119 (4)	0.8243 (5)	0.0706 (15)	
H7A	1.0150	0.0209	0.7971	0.106*	
H7B	0.9554	−0.0505	0.8367	0.106*	
H7C	0.9590	0.0428	0.9058	0.106*	
C8	0.9060 (2)	0.0469 (3)	0.7204 (4)	0.0434 (9)	
C9	0.8629 (2)	0.1226 (3)	0.7219 (4)	0.0497 (10)	
H9	0.8657	0.1654	0.7882	0.060*	
C10	0.8133 (2)	0.1242 (3)	0.6055 (4)	0.0401 (9)	
C11	0.7556 (3)	0.1936 (3)	0.5615 (5)	0.0584 (12)	
H11A	0.7376	0.1834	0.4709	0.088*	
H11B	0.7792	0.2513	0.5699	0.088*	
H11C	0.7129	0.1909	0.6154	0.088*	
C12	0.5787 (2)	0.0682 (3)	0.5086 (4)	0.0376 (8)	
C13	0.5506 (2)	0.0867 (2)	0.3666 (4)	0.0387 (9)	
C14	0.4786 (2)	0.1229 (3)	0.3322 (5)	0.0543 (11)	
H14	0.4470	0.1401	0.3971	0.065*	
C15	0.4546 (3)	0.1331 (3)	0.2014 (5)	0.0624 (13)	
H15	0.4058	0.1558	0.1766	0.075*	
C16	0.5029 (2)	0.1095 (3)	0.1070 (5)	0.0558 (11)	
H16	0.4871	0.1143	0.0175	0.067*	
C17	0.5761 (2)	0.0784 (3)	0.1492 (4)	0.0417 (9)	
C18	0.6004 (3)	0.1738 (3)	−0.1168 (5)	0.0749 (16)	
H18A	0.6331	0.2046	−0.1733	0.112*	
H18B	0.5884	0.2124	−0.0460	0.112*	
H18C	0.5536	0.1564	−0.1668	0.112*	
C19	0.6413 (3)	0.0926 (3)	−0.0609 (4)	0.0540 (11)	
C20	0.6981 (3)	0.0444 (3)	−0.1085 (5)	0.0657 (14)	
H20	0.7179	0.0526	−0.1898	0.079*	
C21	0.7219 (3)	−0.0198 (3)	−0.0151 (4)	0.0567 (12)	
C22	0.7836 (3)	−0.0884 (4)	−0.0240 (5)	0.0836 (17)	
H22A	0.8261	−0.0752	0.0394	0.125*	
H22B	0.8010	−0.0879	−0.1112	0.125*	
H22C	0.7633	−0.1461	−0.0058	0.125*	

### Atomic displacement parameters (Å^2^)

**Table:**

	*U*^11^	*U*^22^	*U*^33^	*U*^12^	*U*^13^	*U*^23^
La1	0.02153 (12)	0.03166 (13)	0.02925 (12)	0.00294 (8)	0.00059 (8)	−0.00108 (8)
N1	0.0216 (14)	0.0310 (15)	0.0276 (14)	0.0017 (11)	0.0031 (11)	0.0041 (12)
N2	0.0269 (15)	0.0347 (15)	0.0346 (16)	−0.0006 (12)	−0.0041 (12)	−0.0010 (13)
N3	0.0275 (15)	0.0330 (16)	0.0408 (17)	0.0017 (12)	−0.0058 (13)	−0.0021 (13)
N4	0.0287 (16)	0.0377 (16)	0.0386 (17)	0.0042 (13)	−0.0018 (13)	0.0012 (13)
N5	0.046 (2)	0.057 (2)	0.0322 (17)	0.0051 (16)	−0.0084 (15)	0.0046 (15)
N6	0.046 (2)	0.068 (2)	0.0322 (17)	0.0118 (17)	0.0012 (15)	0.0004 (16)
N7	0.0322 (18)	0.055 (2)	0.060 (2)	−0.0042 (16)	−0.0058 (16)	0.0234 (18)
O1	0.0303 (14)	0.0451 (16)	0.0511 (16)	0.0081 (11)	−0.0078 (11)	−0.0149 (12)
O2	0.0428 (15)	0.0388 (15)	0.0513 (16)	0.0042 (12)	0.0063 (12)	−0.0164 (13)
O3	0.0309 (14)	0.0502 (16)	0.0362 (14)	0.0111 (11)	0.0028 (11)	−0.0030 (11)
O4	0.0382 (16)	0.074 (2)	0.0579 (18)	0.0116 (14)	0.0128 (14)	−0.0184 (16)
O5	0.0420 (16)	0.0489 (17)	0.0589 (18)	0.0097 (13)	0.0025 (13)	0.0137 (14)
O6	0.0377 (15)	0.0482 (18)	0.0587 (18)	0.0069 (12)	0.0092 (13)	0.0159 (14)
O7	0.0435 (19)	0.080 (3)	0.150 (4)	−0.0069 (18)	0.011 (2)	0.064 (3)
O8	0.0298 (14)	0.0544 (18)	0.076 (2)	−0.0034 (12)	0.0101 (13)	−0.0254 (15)
O9	0.0365 (15)	0.0539 (17)	0.0491 (16)	0.0021 (12)	0.0020 (12)	0.0141 (13)
O10	0.071 (2)	0.065 (2)	0.092 (3)	0.0011 (18)	−0.013 (2)	−0.0078 (19)
C1	0.0314 (19)	0.035 (2)	0.0331 (19)	−0.0008 (15)	0.0071 (15)	−0.0003 (16)
C2	0.0265 (17)	0.0276 (18)	0.0375 (19)	0.0006 (13)	0.0066 (14)	0.0032 (14)
C3	0.031 (2)	0.037 (2)	0.058 (3)	0.0095 (16)	0.0051 (18)	−0.0014 (18)
C4	0.0286 (19)	0.044 (2)	0.064 (3)	0.0105 (17)	−0.0001 (18)	0.005 (2)
C5	0.0275 (18)	0.043 (2)	0.047 (2)	−0.0014 (16)	−0.0042 (16)	0.0060 (17)
C6	0.0243 (17)	0.0311 (18)	0.0324 (18)	−0.0037 (14)	0.0020 (14)	0.0044 (14)
C7	0.058 (3)	0.110 (4)	0.040 (2)	0.013 (3)	−0.016 (2)	−0.010 (3)
C8	0.034 (2)	0.061 (3)	0.035 (2)	0.0005 (18)	−0.0042 (16)	−0.0093 (18)
C9	0.044 (2)	0.058 (3)	0.046 (2)	−0.002 (2)	−0.0007 (19)	−0.025 (2)
C10	0.034 (2)	0.039 (2)	0.047 (2)	−0.0022 (16)	0.0020 (17)	−0.0086 (17)
C11	0.055 (3)	0.041 (2)	0.077 (3)	0.009 (2)	−0.006 (2)	−0.021 (2)
C12	0.030 (2)	0.037 (2)	0.046 (2)	0.0014 (16)	0.0061 (17)	−0.0076 (17)
C13	0.0272 (18)	0.036 (2)	0.052 (2)	0.0031 (15)	−0.0005 (16)	−0.0022 (17)
C14	0.035 (2)	0.056 (3)	0.072 (3)	0.0158 (19)	0.004 (2)	0.004 (2)
C15	0.034 (2)	0.071 (3)	0.080 (3)	0.019 (2)	−0.010 (2)	0.015 (3)
C16	0.044 (2)	0.064 (3)	0.055 (3)	0.008 (2)	−0.017 (2)	0.012 (2)
C17	0.038 (2)	0.044 (2)	0.042 (2)	0.0004 (17)	−0.0060 (17)	0.0018 (17)
C18	0.101 (4)	0.068 (3)	0.052 (3)	−0.006 (3)	−0.013 (3)	0.016 (3)
C19	0.066 (3)	0.060 (3)	0.034 (2)	−0.008 (2)	−0.005 (2)	0.000 (2)
C20	0.086 (4)	0.078 (4)	0.034 (2)	−0.013 (3)	0.007 (2)	0.002 (2)
C21	0.063 (3)	0.071 (3)	0.037 (2)	0.003 (2)	0.009 (2)	−0.012 (2)
C22	0.087 (4)	0.107 (5)	0.061 (3)	0.023 (4)	0.028 (3)	−0.005 (3)

### Geometric parameters (Å, °)

**Table:**

La1—O1	2.452 (2)	C3—C4	1.384 (5)
La1—O3	2.482 (2)	C3—H3	0.9300
La1—O8	2.609 (3)	C4—C5	1.379 (5)
La1—O6	2.630 (3)	C4—H4	0.9300
La1—O9	2.659 (3)	C5—C6	1.392 (5)
La1—O5	2.676 (3)	C5—H5	0.9300
La1—N3	2.688 (3)	C7—C8	1.495 (6)
La1—N1	2.709 (3)	C7—H7A	0.9600
La1—N4	2.727 (3)	C7—H7B	0.9600
La1—N6	2.811 (3)	C7—H7C	0.9600
N1—C6	1.331 (4)	C8—C9	1.363 (6)
N1—C2	1.347 (4)	C9—C10	1.405 (5)
N2—C8	1.378 (5)	C9—H9	0.9300
N2—N3	1.386 (4)	C10—C11	1.491 (6)
N2—C6	1.413 (5)	C11—H11A	0.9600
N3—C10	1.325 (5)	C11—H11B	0.9600
N4—C17	1.331 (5)	C11—H11C	0.9600
N4—C13	1.344 (5)	C12—C13	1.510 (5)
N5—C19	1.369 (5)	C13—C14	1.383 (5)
N5—N6	1.382 (5)	C14—C15	1.369 (7)
N5—C17	1.406 (5)	C14—H14	0.9300
N6—C21	1.329 (6)	C15—C16	1.373 (7)
N7—O7	1.218 (4)	C15—H15	0.9300
N7—O5	1.263 (4)	C16—C17	1.392 (5)
N7—O6	1.275 (4)	C16—H16	0.9300
O1—C1	1.271 (4)	C18—C19	1.501 (7)
O2—C1	1.234 (4)	C18—H18A	0.9600
O3—C12	1.266 (4)	C18—H18B	0.9600
O4—C12	1.238 (4)	C18—H18C	0.9600
O8—H8B	0.8499	C19—C20	1.346 (7)
O8—H8C	0.8500	C20—C21	1.393 (7)
O9—H9B	0.8500	C20—H20	0.9300
O9—H9C	0.8499	C21—C22	1.496 (7)
O10—H10C	0.8501	C22—H22A	0.9600
O10—H10D	0.8499	C22—H22B	0.9600
C1—C2	1.528 (5)	C22—H22C	0.9600
C2—C3	1.375 (5)		
			
O1—La1—O3	138.56 (9)	O2—C1—C2	118.4 (3)
O1—La1—O8	70.21 (8)	O1—C1—C2	115.8 (3)
O3—La1—O8	76.27 (9)	N1—C2—C3	122.8 (3)
O1—La1—O6	80.56 (9)	N1—C2—C1	115.0 (3)
O3—La1—O6	139.19 (9)	C3—C2—C1	122.2 (3)
O8—La1—O6	142.37 (9)	C2—C3—C4	118.1 (4)
O1—La1—O9	84.97 (9)	C2—C3—H3	121.0
O3—La1—O9	62.33 (8)	C4—C3—H3	121.0
O8—La1—O9	73.66 (9)	C5—C4—C3	120.3 (3)
O6—La1—O9	127.78 (8)	C5—C4—H4	119.9
O1—La1—O5	123.50 (10)	C3—C4—H4	119.9
O3—La1—O5	97.58 (9)	C4—C5—C6	117.7 (3)
O8—La1—O5	136.54 (9)	C4—C5—H5	121.1
O6—La1—O5	47.99 (8)	C6—C5—H5	121.1
O9—La1—O5	141.55 (9)	N1—C6—C5	122.8 (3)
O1—La1—N3	120.50 (8)	N1—C6—N2	114.8 (3)
O3—La1—N3	76.17 (9)	C5—C6—N2	122.3 (3)
O8—La1—N3	144.42 (10)	C8—C7—H7A	109.5
O6—La1—N3	71.41 (10)	C8—C7—H7B	109.5
O9—La1—N3	73.81 (9)	H7A—C7—H7B	109.5
O5—La1—N3	69.44 (9)	C8—C7—H7C	109.5
O1—La1—N1	61.59 (8)	H7A—C7—H7C	109.5
O3—La1—N1	117.58 (8)	H7B—C7—H7C	109.5
O8—La1—N1	117.29 (9)	C9—C8—N2	105.7 (3)
O6—La1—N1	64.12 (8)	C9—C8—C7	128.8 (4)
O9—La1—N1	64.84 (8)	N2—C8—C7	125.4 (4)
O5—La1—N1	103.81 (8)	C8—C9—C10	107.8 (3)
N3—La1—N1	59.02 (8)	C8—C9—H9	126.1
O1—La1—N4	125.28 (8)	C10—C9—H9	126.1
O3—La1—N4	61.02 (9)	N3—C10—C9	109.9 (3)
O8—La1—N4	70.58 (9)	N3—C10—C11	122.1 (3)
O6—La1—N4	111.20 (9)	C9—C10—C11	128.0 (4)
O9—La1—N4	118.00 (9)	C10—C11—H11A	109.5
O5—La1—N4	69.10 (9)	C10—C11—H11B	109.5
N3—La1—N4	113.69 (9)	H11A—C11—H11B	109.5
N1—La1—N4	171.90 (9)	C10—C11—H11C	109.5
O1—La1—N6	77.64 (10)	H11A—C11—H11C	109.5
O3—La1—N6	119.69 (9)	H11B—C11—H11C	109.5
O8—La1—N6	79.60 (11)	O4—C12—O3	125.6 (4)
O6—La1—N6	71.22 (10)	O4—C12—C13	118.7 (3)
O9—La1—N6	151.80 (10)	O3—C12—C13	115.7 (3)
O5—La1—N6	66.17 (10)	N4—C13—C14	122.1 (4)
N3—La1—N6	134.28 (10)	N4—C13—C12	116.0 (3)
N1—La1—N6	122.64 (9)	C14—C13—C12	121.9 (4)
N4—La1—N6	58.94 (9)	C15—C14—C13	119.1 (4)
C6—N1—C2	118.3 (3)	C15—C14—H14	120.4
C6—N1—La1	124.0 (2)	C13—C14—H14	120.4
C2—N1—La1	117.3 (2)	C14—C15—C16	119.6 (4)
C8—N2—N3	110.6 (3)	C14—C15—H15	120.2
C8—N2—C6	131.7 (3)	C16—C15—H15	120.2
N3—N2—C6	117.7 (3)	C15—C16—C17	118.0 (4)
C10—N3—N2	106.0 (3)	C15—C16—H16	121.0
C10—N3—La1	129.4 (2)	C17—C16—H16	121.0
N2—N3—La1	119.2 (2)	N4—C17—C16	123.1 (4)
C17—N4—C13	117.9 (3)	N4—C17—N5	115.2 (3)
C17—N4—La1	124.0 (2)	C16—C17—N5	121.7 (4)
C13—N4—La1	116.9 (2)	C19—C18—H18A	109.5
C19—N5—N6	111.5 (4)	C19—C18—H18B	109.5
C19—N5—C17	129.2 (4)	H18A—C18—H18B	109.5
N6—N5—C17	119.3 (3)	C19—C18—H18C	109.5
C21—N6—N5	104.7 (3)	H18A—C18—H18C	109.5
C21—N6—La1	130.9 (3)	H18B—C18—H18C	109.5
N5—N6—La1	114.8 (2)	C20—C19—N5	105.4 (4)
O7—N7—O5	122.4 (4)	C20—C19—C18	129.8 (4)
O7—N7—O6	121.1 (4)	N5—C19—C18	124.7 (4)
O5—N7—O6	116.5 (3)	C19—C20—C21	108.3 (4)
C1—O1—La1	129.3 (2)	C19—C20—H20	125.9
C12—O3—La1	129.5 (2)	C21—C20—H20	125.9
N7—O5—La1	96.7 (2)	N6—C21—C20	110.2 (4)
N7—O6—La1	98.6 (2)	N6—C21—C22	122.2 (4)
La1—O8—H8B	111.1	C20—C21—C22	127.7 (5)
La1—O8—H8C	111.3	C21—C22—H22A	109.5
H8B—O8—H8C	109.2	C21—C22—H22B	109.5
La1—O9—H9B	110.3	H22A—C22—H22B	109.5
La1—O9—H9C	110.2	C21—C22—H22C	109.5
H9B—O9—H9C	108.6	H22A—C22—H22C	109.5
H10C—O10—H10D	107.0	H22B—C22—H22C	109.5
O2—C1—O1	125.8 (3)		
			
O1—La1—N1—C6	−179.1 (3)	N4—La1—O3—C12	5.2 (3)
O3—La1—N1—C6	−46.4 (3)	N6—La1—O3—C12	−0.6 (3)
O8—La1—N1—C6	−134.5 (2)	O7—N7—O5—La1	−175.3 (4)
O6—La1—N1—C6	87.7 (3)	O6—N7—O5—La1	4.1 (3)
O9—La1—N1—C6	−80.9 (2)	O1—La1—O5—N7	−33.0 (3)
O5—La1—N1—C6	60.0 (3)	O3—La1—O5—N7	152.8 (2)
N3—La1—N1—C6	4.7 (2)	O8—La1—O5—N7	−129.2 (2)
N4—La1—N1—C6	31.5 (7)	O6—La1—O5—N7	−2.4 (2)
N6—La1—N1—C6	130.3 (2)	O9—La1—O5—N7	98.7 (2)
O1—La1—N1—C2	7.7 (2)	N3—La1—O5—N7	80.7 (2)
O3—La1—N1—C2	140.5 (2)	N1—La1—O5—N7	31.9 (2)
O8—La1—N1—C2	52.3 (2)	N4—La1—O5—N7	−152.2 (2)
O6—La1—N1—C2	−85.4 (2)	N6—La1—O5—N7	−88.0 (2)
O9—La1—N1—C2	106.0 (2)	O7—N7—O6—La1	175.2 (4)
O5—La1—N1—C2	−113.1 (2)	O5—N7—O6—La1	−4.2 (4)
N3—La1—N1—C2	−168.4 (3)	O1—La1—O6—N7	156.9 (2)
N4—La1—N1—C2	−141.6 (6)	O3—La1—O6—N7	−37.2 (3)
N6—La1—N1—C2	−42.8 (3)	O8—La1—O6—N7	117.9 (2)
C8—N2—N3—C10	1.6 (4)	O9—La1—O6—N7	−127.1 (2)
C6—N2—N3—C10	−175.5 (3)	O5—La1—O6—N7	2.4 (2)
C8—N2—N3—La1	−155.0 (3)	N3—La1—O6—N7	−76.3 (2)
C6—N2—N3—La1	27.9 (4)	N1—La1—O6—N7	−140.2 (2)
O1—La1—N3—C10	−170.6 (3)	N4—La1—O6—N7	32.6 (2)
O3—La1—N3—C10	−31.9 (3)	N6—La1—O6—N7	76.8 (2)
O8—La1—N3—C10	−72.1 (4)	La1—O1—C1—O2	−172.2 (3)
O6—La1—N3—C10	122.9 (3)	La1—O1—C1—C2	8.3 (5)
O9—La1—N3—C10	−96.7 (3)	C6—N1—C2—C3	−0.3 (5)
O5—La1—N3—C10	71.8 (3)	La1—N1—C2—C3	173.2 (3)
N1—La1—N3—C10	−166.7 (4)	C6—N1—C2—C1	179.2 (3)
N4—La1—N3—C10	17.3 (3)	La1—N1—C2—C1	−7.3 (4)
N6—La1—N3—C10	86.3 (3)	O2—C1—C2—N1	−179.0 (3)
O1—La1—N3—N2	−20.2 (3)	O1—C1—C2—N1	0.6 (4)
O3—La1—N3—N2	118.4 (3)	O2—C1—C2—C3	0.4 (5)
O8—La1—N3—N2	78.3 (3)	O1—C1—C2—C3	−180.0 (3)
O6—La1—N3—N2	−86.7 (2)	N1—C2—C3—C4	−0.2 (6)
O9—La1—N3—N2	53.7 (2)	C1—C2—C3—C4	−179.6 (3)
O5—La1—N3—N2	−137.8 (3)	C2—C3—C4—C5	−0.2 (6)
N1—La1—N3—N2	−16.3 (2)	C3—C4—C5—C6	1.0 (6)
N4—La1—N3—N2	167.7 (2)	C2—N1—C6—C5	1.2 (5)
N6—La1—N3—N2	−123.4 (2)	La1—N1—C6—C5	−171.9 (3)
O1—La1—N4—C17	−44.0 (3)	C2—N1—C6—N2	179.8 (3)
O3—La1—N4—C17	−175.1 (3)	La1—N1—C6—N2	6.8 (4)
O8—La1—N4—C17	−90.4 (3)	C4—C5—C6—N1	−1.5 (5)
O6—La1—N4—C17	49.4 (3)	C4—C5—C6—N2	179.9 (3)
O9—La1—N4—C17	−148.8 (3)	C8—N2—C6—N1	161.2 (4)
O5—La1—N4—C17	73.0 (3)	N3—N2—C6—N1	−22.4 (4)
N3—La1—N4—C17	127.6 (3)	C8—N2—C6—C5	−20.2 (6)
N1—La1—N4—C17	102.6 (6)	N3—N2—C6—C5	156.2 (3)
N6—La1—N4—C17	−1.1 (3)	N3—N2—C8—C9	−1.4 (4)
O1—La1—N4—C13	123.1 (3)	C6—N2—C8—C9	175.2 (4)
O3—La1—N4—C13	−8.1 (2)	N3—N2—C8—C7	175.9 (4)
O8—La1—N4—C13	76.6 (3)	C6—N2—C8—C7	−7.5 (7)
O6—La1—N4—C13	−143.6 (3)	N2—C8—C9—C10	0.7 (5)
O9—La1—N4—C13	18.3 (3)	C7—C8—C9—C10	−176.5 (5)
O5—La1—N4—C13	−120.0 (3)	N2—N3—C10—C9	−1.1 (4)
N3—La1—N4—C13	−65.3 (3)	La1—N3—C10—C9	152.2 (3)
N1—La1—N4—C13	−90.3 (6)	N2—N3—C10—C11	178.7 (4)
N6—La1—N4—C13	166.0 (3)	La1—N3—C10—C11	−28.0 (6)
C19—N5—N6—C21	−2.3 (5)	C8—C9—C10—N3	0.3 (5)
C17—N5—N6—C21	177.8 (4)	C8—C9—C10—C11	−179.5 (4)
C19—N5—N6—La1	147.9 (3)	La1—O3—C12—O4	177.9 (3)
C17—N5—N6—La1	−32.0 (4)	La1—O3—C12—C13	−1.9 (5)
O1—La1—N6—C21	−58.1 (4)	C17—N4—C13—C14	−0.9 (6)
O3—La1—N6—C21	162.6 (4)	La1—N4—C13—C14	−168.8 (3)
O8—La1—N6—C21	−129.9 (4)	C17—N4—C13—C12	178.5 (3)
O6—La1—N6—C21	26.0 (4)	La1—N4—C13—C12	10.6 (4)
O9—La1—N6—C21	−111.3 (4)	O4—C12—C13—N4	173.7 (4)
O5—La1—N6—C21	77.5 (4)	O3—C12—C13—N4	−6.5 (5)
N3—La1—N6—C21	62.7 (4)	O4—C12—C13—C14	−7.0 (6)
N1—La1—N6—C21	−14.1 (5)	O3—C12—C13—C14	172.9 (4)
N4—La1—N6—C21	156.6 (4)	N4—C13—C14—C15	3.3 (7)
O1—La1—N6—N5	161.5 (3)	C12—C13—C14—C15	−176.0 (4)
O3—La1—N6—N5	22.2 (3)	C13—C14—C15—C16	−1.8 (7)
O8—La1—N6—N5	89.7 (3)	C14—C15—C16—C17	−1.9 (7)
O6—La1—N6—N5	−114.4 (3)	C13—N4—C17—C16	−3.1 (6)
O9—La1—N6—N5	108.3 (3)	La1—N4—C17—C16	163.9 (3)
O5—La1—N6—N5	−62.9 (3)	C13—N4—C17—N5	179.1 (3)
N3—La1—N6—N5	−77.7 (3)	La1—N4—C17—N5	−13.9 (5)
N1—La1—N6—N5	−154.5 (2)	C15—C16—C17—N4	4.5 (7)
N4—La1—N6—N5	16.2 (3)	C15—C16—C17—N5	−177.9 (4)
O3—La1—O1—C1	−109.3 (3)	C19—N5—C17—N4	−148.7 (4)
O8—La1—O1—C1	−147.2 (3)	N6—N5—C17—N4	31.1 (5)
O6—La1—O1—C1	56.9 (3)	C19—N5—C17—C16	33.4 (7)
O9—La1—O1—C1	−72.8 (3)	N6—N5—C17—C16	−146.7 (4)
O5—La1—O1—C1	79.4 (3)	N6—N5—C19—C20	2.3 (5)
N3—La1—O1—C1	−4.9 (3)	C17—N5—C19—C20	−177.8 (4)
N1—La1—O1—C1	−8.7 (3)	N6—N5—C19—C18	−173.9 (4)
N4—La1—O1—C1	166.2 (3)	C17—N5—C19—C18	6.0 (7)
N6—La1—O1—C1	129.6 (3)	N5—C19—C20—C21	−1.4 (5)
O1—La1—O3—C12	−106.5 (3)	C18—C19—C20—C21	174.5 (5)
O8—La1—O3—C12	−70.0 (3)	N5—N6—C21—C20	1.3 (5)
O6—La1—O3—C12	94.7 (3)	La1—N6—C21—C20	−141.9 (4)
O9—La1—O3—C12	−148.5 (3)	N5—N6—C21—C22	−178.8 (5)
O5—La1—O3—C12	66.2 (3)	La1—N6—C21—C22	37.9 (7)
N3—La1—O3—C12	132.8 (3)	C19—C20—C21—N6	0.0 (6)
N1—La1—O3—C12	176.2 (3)	C19—C20—C21—C22	−179.8 (5)

### Hydrogen-bond geometry (Å, °)

**Table:**

*D*—H···*A*	*D*—H	H···*A*	*D*···*A*	*D*—H···*A*
O8—H8B···O10^i^	0.85	2.13	2.918 (5)	154
O8—H8C···O4^ii^	0.85	1.91	2.713 (4)	158
O9—H9B···O2^iii^	0.85	1.96	2.731 (4)	151
O9—H9B···N1	0.85	2.46	2.878 (4)	112
O10—H10C···N6^iv^	0.85	2.49	3.156 (5)	136
O10—H10D···O9^v^	0.85	2.24	2.977 (5)	146
O10—H10D···O1^iv^	0.85	2.46	2.913 (4)	114

Symmetry codes: (i) *x*, *y*−1, *z*; (ii) −*x*+1, −*y*, −*z*+1; (iii) *x*, −*y*−1/2, *z*+1/2; (iv) *x*, *y*+1, *z*; (v) *x*, −*y*+1/2, *z*−1/2.
